# Disrupted Microbiota of Colon Results in Worse Immunity and Metabolism in Low-Birth-Weight Jinhua Newborn Piglets

**DOI:** 10.3390/microorganisms12071371

**Published:** 2024-07-04

**Authors:** Jiaheng Li, Zeou Wei, Fangfang Lou, Xiaojun Zhang, Jiujun Duan, Chengzeng Luo, Xujin Hu, Pingguang Tu, Lei Liu, Ruqing Zhong, Liang Chen, Xizhong Du, Hongfu Zhang

**Affiliations:** 1 Institute of Animal Husbandry and Veterinary Medicine, Jinhua Academy of Agricultural Sciences, Jinhua 321011, China; jiaheng.li@uqconnect.edu.au (J.L.); m13566994867@163.com (F.L.); zzz_502@163.com (X.Z.); huxujin@sohu.com (X.H.); tupg912@163.com (P.T.); dxz5418@163.com (X.D.); 2 State Key Laboratory of Animal Nutrition and Feeding, Institute of Animal Science, Chinese Academy of Agricultural Sciences, Beijing 100193, China; zeou.wei@ucdconnect.ie (Z.W.); d18653977236@163.com (J.D.); chengzengluo@163.com (C.L.); liulei02@caas.cn (L.L.); chenliang01@caas.cn (L.C.); zhanghongfu@caas.cn (H.Z.); 3 Precision Livestock and Nutrition Unit, Gembloux Agro-Bio Tech, TERRA Teaching and Research Centre, Liège University, Passage des Déportés 2, 5030 Gembloux, Belgium; 4 School of Agriculture and Food Science, University College Dublin, Belfeld, D04 V1W8 Dublin, Ireland

**Keywords:** Jinhua newborn piglet, low-birth-weight, microbiome, transcriptome, glucose metabolism, immunity, short chain fatty acids

## Abstract

The Jinhua pig is well known in China due to its delicious meat. However, because of large litter size, low birth weight always happens. This experiment used this breed as a model to research bacterial evidence leading to growth restriction and provide a possible solution linked to probiotics. In this experiment, the differences in organs indexes, colonic morphology, short chain fatty acid (SCFA) concentrations, microbiome, and transcriptome were detected between piglets in the standard-birth-weight group (SG) and low-birth-weight group (LG) to find potential evidence leading to low birth weight. We found that LG piglets had a lower liver index (*p* < 0.05), deeper colonic crypt depth (*p* < 0.05), fewer goblet cells (*p* < 0.05), and more inflammatory factor infiltration. In addition, differentially expressed genes (DEGs) were mainly enriched in B-cell immunity and glucose metabolism, and LG piglets had lower concentrations of SCFAs, especially butyrate and isobutyrate (*p* < 0.05). Finally, most of the significantly differentially abundant microbes were fewer in LG piglets, which affected DEG expressions and SCFA concentrations further resulting in worse energy metabolism and immunity. In conclusion, colonic disrupted microbiota may cause worse glucose metabolism, immunity, and SCFA production in LG piglets, and beneficial microbes colonized in SG piglets may benefit these harmful changes.

## 1. Introduction

The Jinhua pig, also known as Jinhua two-head black pig, is one of the four famous native breeds in China because of its famous products named Jinhua ham. It has great breeding potential, such that the average number of live piglets for primiparous sows can reach to 10, and the average number of live piglets for sows with more than three litters is 13 [1]. However, large litter sizes are always accompanied by more low-birth-weight piglets such as intrauterine growth restriction (IUGR) piglets [2], which significantly affects the growth performance and health of piglets. Therefore, for one thing, preventing or addressing growth restriction in weaning piglets will directly increase the production of Jinhua pigs. For another, researching the bacterial reason why low birth weight happens in this susceptible breed can also help us to solve several related children’s diseases.

Investigating growth restriction reasons, poor nutrient metabolism and health situation should be the key factors [3]. For example, glucose as the direct energy nutrient is necessary for animal survival, and glucose metabolism is always impaired, resulting in liver metabolism damage in IUGR piglets [4]. Meanwhile, short chain fatty acids (SCFAs) can provide energy to animals and maintain gut barrier integrity to protect the intestine from inflammation [5,6]. Fewer productions of SCFAs always exists in low-birth-weight piglets [7]. Therefore, finding the factors affecting glucose or SCFA metabolism in low-birth-weight piglets could become a target spot to address growth restriction. In addition, poor immunity always occurs in low-birth-weight piglets [3]. Various observations have found that IUGR piglets have worse immunity [8,9]. B-lymphocytes play a key role in humoral immunity of the adaptive immune response. B-cells can protect hosts against invasive pathogens via the production of antigen-specific immunoglobulin (Ig) [10]. Furthermore, B-cell immunity can also affect other immunity processes such as T-cell immunity by producing IL-10 [11]. Therefore, finding the cause leading to abnormal B-cell immunity function could benefit low-birth-weight piglets’ production.

Microbes can be seen as another organ for the host, as it can maintain immunity and metabolism to act against pathogens [12]. A stable gut microbial community will support animals to survive, with most of the microbes significantly influencing nutrient metabolism, especially SCFA production, by their products [13]. Meanwhile, they can maintain the immunity system of the host by various metabolites [14]. Moreover, low-birth-weight always happens when growth or development of the mammalian embryo/fetus or organs is impaired during pregnancy, which could directly affect the microbiota of the offspring [15]. Various studies have found that the microbiota was changed when low birth weight occurred [15,16,17]. Therefore, intestinal microbiota could be a potential factor affecting the nutrient metabolism and immunity of low-birth-weight piglets.

However, there are a few studies focused on the changes in low-birth-weight Jinhua piglets. In this experiment, we investigated the differences in the organ indexes and colonic morphology in low-birth-weight piglets. Then, we combined the colonic SCFA, transcriptome, and microbiome results. Based on the differences and links, we hope, through our findings, to address growth retardation to benefit Jinhua piglets or children through means such as adding probiotics or using fecal microbiota transplantation (FMT) in the host to change the microbiota.

## 2. Materials and Methods

The Animal Ethics Committee of the Institute of Animal Sciences, Chinese Academy of Agricultural Sciences, approved the experimental protocol (Ethics Approval Code: IAS2022-156).

### 2.1. Experimental Piglets and Husbandry Practices

Three pens of newborn Jinhua piglets were fed in the same house and born by similar reproduction performance of sows. Sows were provided food and water ad libitum. All ingredients and composition of diets are presented in Table S1. Fans and a cooling pad were used to automatically control the temperature, humidity, and air flow rate.

### 2.2. Experimental Design

After birth, all piglets were weighed individually. Twelve piglets were randomly selected and divided into two groups, which were the standard-birth-weight group (SG) and low-birth-weight group (LG) based on the birth weight, in which low-birth-weight piglets’ body weight should be lower than 1 kg. All piglets were breastfed for 15 days.

### 2.3. Samples Collocation

On day 15, piglets were weighed individually in the morning. After weighing, they were euthanized by injecting sodium pentobarbital (50 mg/kg BW) and then exsanguinated. After removing and weighing all organs, colonic segment samples of about 4 cm length (10 cm to the ileum) were cut into two parts and separately fixed in Carnot’s solution and 4% paraformaldehyde fixative solution. Thereafter, we collected colonic mucosa and feces and stored them in −80 °C for detecting.

### 2.4. Growth Performance and Organ Index Statistical Analysis

The organ index was defined as dividing organ weight by body weight (mg/g). Shapiro–Wilk test was used to detect the normality of data. Based on the normality result, the Student’s *t* test or Mann–Whitney test was used to determine statistical significance using IBM SPSS Statistics software version 21.

### 2.5. Histological Evaluation

After dehydrating and clearing, colon samples were embedded in paraffin wax. Thereafter, we cut two 5 mm sections and deparaffinized them in xylene. After rehydrating, Alcian blue-periodic acid-Schif (AB-PAS) and hematoxylin and eosin (HE) were used to stain sections. Leica Application Suite X (Leica Microsystems, Wetzlar, Germany) was used to scan slides under a microscope at 80× magnification, and for the goblets cells numbers and crypt depth, we randomly chose 10 crypts from different parts of each sample. For the AB-PAS section, the number of goblet cells was counted manually in each crypt. For the HE section, the crypt depth was measured from the bottom of the crypt to the top of each crypt. In the analyzing process, we analyzed the normality of data firstly by the Shapiro–Wilk test, and based on the normality, we chose the Mann–Whitney test or Student’s *t* test to determine statistical significance. All analyzing processes used IBM SPSS Statistics software version 21.

### 2.6. Short Chain Fatty Acid (SCFA) Determination

A method was followed according to a previous study [18], using ultrapure water to extract digesta samples from colon (around 0.5 g). Then, samples were centrifugated at 10,000× *g*. Thereafter, metaphosphoric acid (25%, *w*/*v*) was used to mix samples, in which the amounts of extracts were 9 times more than acid. In addition, the mixture was centrifugated at 12,000× *g*, and the supernatant was filtered through the 0.45 µm Milled-LG filter (Millipore, Billerica, MA, USA). Finally, Agilent 7890 N gas chromatograph (Agilent, Santa Clara, CA, USA) was used to analyze the relative concentrations of SCFAs. Normality of data was determined by the Shapiro–Wilk test. Statistical significance was determined by Student’s *t* test or the Mann–Whitney test based on the normality result using IBM SPSS Statistics software version 21.

### 2.7. RNA Extraction and Sequencing

Total RNA was isolated using Trizol Reagent, and its concentration, quality, and integrity were determined using the NanoDrop spectrophotometer. Magnetic beads coated with Poly T oligo were then used to purify mRNA from three micrograms of RNA in each sample. The purified mRNA was then fragmented and reverse transcribed into cDNA, followed by adenylation and ligation to Illumina PE adapter oligonucleotides preparing for hybridization. After 15-cycle PCR reactions amplifying cDNA, Agilent high sensitivity DNA assay on a Bioanalyzer 2100 system (Agilent, Santa Clara, CA) was used to purify and quantify the amplified products. Finally, NovaSeq 6000 platform (Illumina, Shanghai, China) was used to sequence the sequencing library.

### 2.8. RNA-Seq Quality Control, Read Mapping, and Filtering

Image files were obtained by sequencing the samples on the platform, which were then subsequently processed by the sequencing platform software (https://www.genescloud.cn (accessed on 2 September 2023)) to generate original data in FASTQ format (raw data). Thereafter, the sequencing data were filtered using Cutadapt (version 1.15) to acquire high quality sequence clean data for further analysis. A genome website was used to download the genome reference and gene annotation files. HISAT2 v2.0.5 was used to map the filtered reads to the reference genome. The raw data were deposited into the NCBI Sequence Read Archive (SRA) database with Accession Number PRJNA1018252.

### 2.9. RNA-Seq Differential Expression and Enrichment Analysis

The read count values were compared using HTSeq (version 0.9.1) to determine the original expression of genes. Fragments Per Kilobase of transcript per Million mapped reads (FPKMs) were utilized to standardize the expression. Based on the conditions including a fold change of 2 or greater (|log2FoldChange| > 1) and a *p*-value less than 0.05, the differential expression of genes was analyzed using DESeq2 (version 3.17). The principal analysis (PCA) result was plotted using Factoextra package (version 1.0.7). The fold change in differentially expressed genes (DEGs) was plotted by the package pheatmap (Version 1.0.12) in R (Version 4.3.1). All the genes to Terms were mapped in the gene ontology database, and the numbers of differentially enriched genes were calculated in each Term. GO enrichment analysis was performed using topGO, and the *p*-value was calculated by the hypergeometric distribution method (the standard of significant enrichment is a *p*-value < 0.05). Then, the GO term with significantly enriched differential genes was found to determine the main biological functions performed by differential genes. Finally, we used ClusterProfiler (version 3.4.4) to subject the enrichment analysis of the KEGG pathway of differentially expressed genes, with a focus on significant enrichment pathways with a *p*-value < 0.05.

### 2.10. 16S rRNA Extraction and Sequencing

E.Z.N.A.^®^ soil DNA kit (Omega Bio-tek, Norcross, GA, USA) was used to extract the DNA from colonic digesta and mucosa samples, in which the V3–V4 region of bacterial 16S rRNA genes was amplified using the T100 Thermal Cycler PCR thermocycler (BIO-RAD, Hercules, CA, USA) and the following primers: 338F (5′-ACTCCTACGGGAGGCAGCAG-3′) and 806R (5′-GGACTACHVGGGTWTCTAAT-3′).

The purified amplicons were then combined in equal amounts and subjected to paired-end sequencing using the Illumina PE300 platform (Illumina, San Diego, CA, USA), following the standard protocols provided by Majorbio Bio-Pharm Technology Co., Ltd. (Shanghai, China). The resulting raw data were deposited into the NCBI Sequence Read Archive (SRA) database with the Accession Number: PRJNA1017154.

### 2.11. Colonic Microbiome Data Processing

Fastp (version 0.19.6) and FLASH (version 1.2.7) were used to quality-filter and merge resulting sequences after demultiplexing. DADA2 (v. 1.18) package in R was used to denoise the high-quality sequences to obtain amplicon sequence variants (ASVs). Based on the SILVA 16S rRNA database (v138), taxonomic assignment of ASVs was performed using the Naive bayes consensus taxonomy classifier implemented in Qiime2. Then, to minimize the impact of sequencing depth on subsequent analysis, the number of 16S rRNA gene sequences was rarefied to the minimum number of sequences present in any sample.

### 2.12. Colonic Microbiome Statistical Analysis

Majorbio Cloud platform (https://cloud.majorbio.com (Accessed 2 Sept. 2023)) was used to conduct a bioinformatic analysis of the colonic microbiota. Mothur (version 1.30.1) was used to determine alpha diversity indices, and the Wilcoxon rank-sum test was used to calculate statistical significance. R (Version 4.3.1) was used to analyze beta diversity, in which the vegan package (version 2.6-4) was used to plot the principal analysis (PCA) and principal coordinate analysis (PCoA). The Wilcoxon rank-sum test was used to assess the percentage of variation explained by the treatment along with its statistical significance. The significantly abundant microbial taxa between piglets in two groups (LDA score > 2, *p* < 0.05) were determined by the linear discriminant analysis effect size (LEfSe).

### 2.13. Correlation Analysis

In all significantly differential enrichment pathways, we selected three pathways which related to glucose metabolism containing nine differentially expressed genes (DEGs) enriched and two pathways which related to B-cell immunity containing 14 DEGs enriched. Meanwhile, all significantly differentially abundant microbes in the top 50 abundant microbes at the Genus level from both colonic mucosa and their contents were selected. Thereafter, we analyzed the relationships between these microbes and DEGs or SCFAs. Package psych (Version 2.3.6) in R (Version 4.3.1) was used to analyze the correlation results, and pheatmap (Version 1.0.12) in R (Version 4.3.1) was used to plot the results.

### 2.14. Validation of DEGs Using qRT-PCR

Tissue/Cell Total RNA Mini Kit (Gene-Better, Beijing, China) was used to isolate total RNA from colonic mucosa. Then, Prime Script RT reagent Kit (Takara, Kusatsu, Shiga, Japan) was used to reverse transcribe 2 μg total mRNA. Then, 1 μL cDNA was mixed with 0.2 µL each of forward and reverse primers (final concentration of 0.2 µM for each primer) (Sangon Biotech, Shanghai, China) (Table S3), 0.2 μL ROX Reference Dye II, 5 μL SYBR Premix Ex Taq II, and 3.4 µL double distilled water to react for qPCR. QuantStudio 7 Flex (Thermofisher, Waltham, MA, USA) was used for amplification and detection as follows: 1. Pre-denaturation stage: at 95 °C for 30 s; 2. PCR stage (40 cycles): denaturation at 95 °C for 10 s, followed by annealing and extension at 60 °C for 30 s; 3. Melt curve: at 95 °C for 15 s, 60 °C for 1 min, and 95 °C for 15 s. The reference gene was *GAPDH* to normalize the gene’s Ct values. The relative gene expression was calculated by 2^−ΔΔCt^ method.

## 3. Results

### 3.1. Low-Birth-Weight Piglets Had Lower Organ Indexes

The body weights of piglets in the two groups are shown in Table S2. The organ indexes are shown in Table 1. There were significant differences in all parameters (*p* < 0.05) except for spleen index (*p* = 0.071), in which the liver index had the lowest *p*-value (*p* = 0.011), among all organ indexes. For all parameters, piglets in the low-birth-weight group (LG) had lower results compared with the standard-birth-weight group (SG).

### 3.2. Low-Birth-Weight Piglets Had Worse Colonic Morphology and Secretory Function

**Figure 1:**
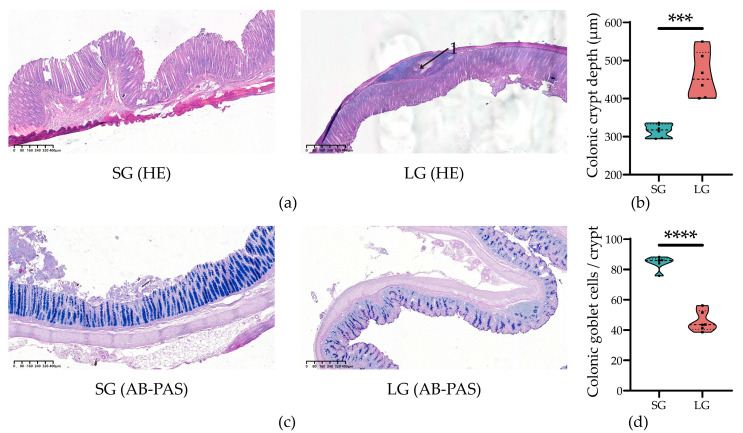
Differences in colonic crypt morphology and goblet cells numbers between SG and LG group piglets. (**a**) Representative images of HE-stained colonic sections. Scale bar = 80 μm. (**b**) The crypt depth of colon. (**c**) Representative images of AB-PAS-stained colonic sections. Scale bar = 80 μm. (**d**) The number of goblet cells in the colon. 1, inflammatory factor infiltration; SG, standard-birth-weight group; LG, low-birth-weight group; HE, hematoxylin and eosin; AB-PAS, alcian blue-periodic acid-Schif; Data are presented from min to max with all points; Normality of data was determined by the Shapiro–Wilk test; statistical significance was determined by Student’s *t* test or Mann–Whitney test based on the normality result; *n* = 6; *** *p* < 0.001 and **** *p* < 0.0001.

The colonic mucus layer can defend against the invasion of pathogens and harmful bacteria as the first barrier. The colonic slides’ result is shown in Figure 1, in which the inflammatory factor infiltration appeared in LG (Figure 1a). Hematoxylin and eosin (HE) (Figure 1a) staining picture shows that the colonic crypt depth (Figure 1b) (*p* < 0.001) was highly significantly reduced in piglets in LG compared with those in SG, and the Alcian blue-periodic acid-Schif (AB-PAS) (Figure 1c) staining picture shows that the number of colonic goblet cells (Figure 1d) (*p* < 0.0001) was highly significantly reduced in piglets in LG compared with those in SG.

### 3.3. Low-Birth-Weight Piglets Have Differential Gene Expression in Colonic Mucosa

**Figure 2:**
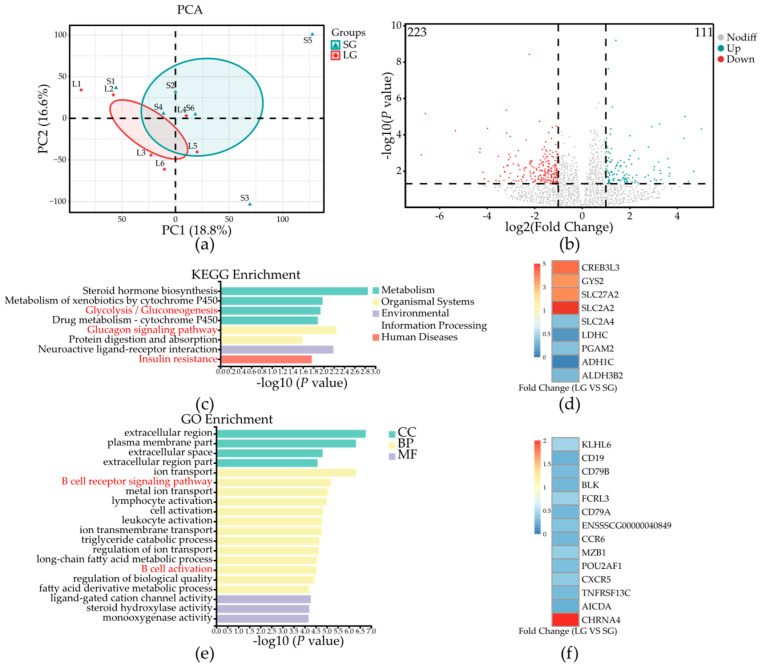
The gene expression in colonic mucosa of piglets in the two groups. (**a**) PCA results of genes in colonic mucosa. (**b**) Volcano plot of DEGs in colonic mucosa (FC > 2, *p* < 0.05) (LG vs. SG). (**c**) KEGG enrichment of DEGs in colonic mucosa. (**d**) The fold change in nine differentially expressed genes enriched in glucose metabolism. (**e**) Go enrichment of DEGs in colonic mucosa. (**f**) The fold change in 14 differentially expressed genes enriched in B-cell immunity system. The focused pathways were marked with red fonts. SG, standard-birth-weight group; LG, low-birth-weight group; DEGs, differentially expressed genes; *n* = 6.

The metabolism and immunity situation of piglets can be explained by differential gene expression. The differential gene expression status is shown in Figure 2. A PCA analysis showed that there was a difference in gene expression between piglets in the two groups (Figure 2a). Specifically, using DESeq2 analysis (Figure 2b) (FC > 2, *p* < 0.05), 111 upregulated genes and 223 downregulated genes were discovered in piglets in LG compared with those in SG.

Based on the KEGG enrichment result, differentially expressed genes (DEGs) were mainly enriched in metabolism and organismal systems. We chose three pathways related to glucose metabolism for the next analysis, which were the glucagon signaling pathway, glycolysis/gluconeogenesis, and insulin resistance (Figure 2c). In the three enrichment pathways, nine DEGs were found, which were *CREB3L3*, *GYS2*, *SLC27A2*, *SLC2A2*, *SLC2A4*, *LDHC*, *PGAM2*, *ADH1C*, and *ALDH3B2*. The expression status of piglets in the two groups is shown in Figure 2d, in which *CREB3L3*, *GYS2*, *SLC27A2*, and *SLC2A2* were upregulated DEGs, and others were downregulated (LG vs. SG).

Go enrichment showed that differentially expressed genes (DEGs) were mainly enriched in the biological process (BP), in which two pathways related to immune system were chosen, which were the B cell receptor signaling pathway and B cell activation (Figure 2e). In these two enrichment pathways, 14 DEGs were found, which were *KLHL6*, *CD19*, *CD79B*, *BLK*, *FCRL3*, *CD79A*, *ENSSSCG00000040849*, *CCR6*, *MZB1*, *POU2AF1*, *CXCR5*, *TNFRSF13C*, *AICDA*, and *CHRNA4*. The expression status of piglets in the two groups is shown in Figure 2f, in which all DEGs except *CHRNA4* were downregulated (LG vs. SG).

### 3.4. Low-Birth-Weight Piglets Had Lower Concentrations of Short Chain Fatty Acids in Colonic Digesta

**Figure 3:**
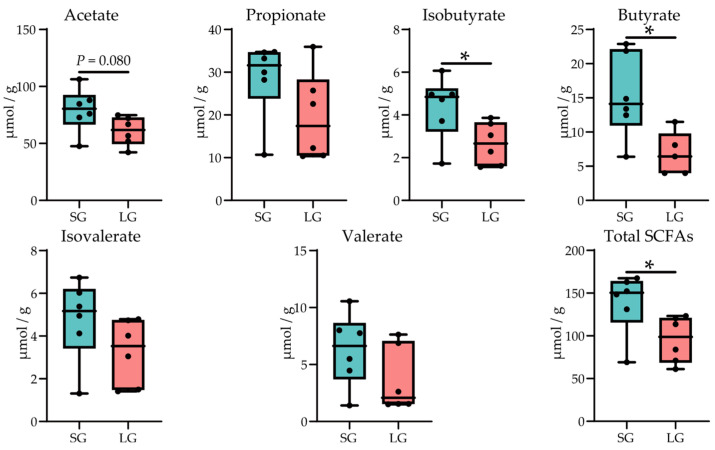
The concentrations of short chain fatty acids in colonic digesta of piglets in two groups. SG, standard-birth-weight group; LG, low-birth-weight group; Data are presented from min to max with all points; Normality of data was determined by the Shapiro–Wilk test; Statistical significance was determined by Student’s *t* test or Wilcoxon rank-sum test based on the normality result; *n* = 6; * *p* < 0.05.

Short chain fatty acids (SCFAs) can be used as an energy source for the improved growth performance of piglets. The concentrations of SCFAs in the colonic contents of piglets in the two groups are shown in Figure 3. In LG, piglets had a significantly lower concentration of isobutyrate, butyrate, and total SCFAs (*p* < 0.05), and the trend showed that piglets in LG had lower concentrations of acetate (*p* = 0.080). For other SCFAs, there was no significant difference (*p* > 0.05).

### 3.5. Low-Birth-Weight Piglets Had Differential Microbes in Colonic Mucosa

**Figure 4:**
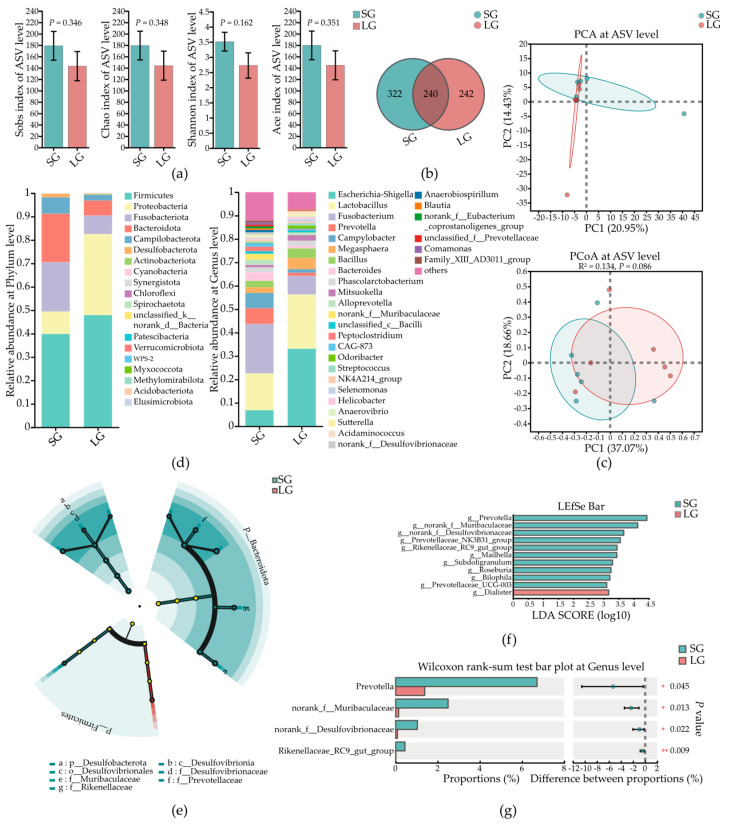
The microbial composition in colonic mucosa of piglets in two groups. (**a**) α-Diversity of microbes in colonic mucosa. (**b**) Venn diagrams of microbes in colonic mucosa. (**c**) PCA and PCoA results of microbes in colonic mucosa. (**d**) Community analysis of microbes in colonic mucosa. (**e**) Cladogram chart of LEfSe result of microbes from Phylum to Family level. (**f**) Bar chart of LEfSe result of microbes at Genus level. (**g**) Top four significantly differentially abundant microbes in colonic mucosa. SG, standard-birth-weight group; LG, low-birth-weight group; LEfSe, linear discriminant analysis effect size; Data are presented as mean ± SE. Statistical significance was determined by the Student’s *t* test (**a**) and Wilcoxon rank-sum test (**g**); *n* = 6; * *p* < 0.05 and ** *p* < 0.01.

The microbes in mucosa can defend against pathogens through their metabolites. Figure 4 shows the microbial composition in colonic mucosa. There was no significant difference in α-diversity (Figure 4a) (*p* > 0.05), and piglets in SG had more ASVs (Figure 4b). For β-diversity, the colonic mucosa microbial composition at ASV level was not significantly different between piglets in the two groups based on PCA and PCoA (*p* = 0.086) results (Figure 4c). Figure 4d shows the specific microbial composition at the Genus and Phylum level. Linear discriminant analysis effect size (LEfSe) analysis was used to identify the dominant microbial taxa in piglets in two groups (Figure 4e,f). In Top 50 abundance of microbes at Genus level, the abundances of four microbial taxa were found significantly decreased in LG, which were *Prevotella*, *norank_f_Muribaculaceae*, *norank_f_Desulfovibrionaceae*, and *Rikenellaceae_RC9_gut_group* (Figure 4g).

### 3.6. Low-Birth-Weight Piglets Had Differential Microbes in Colonic Contents

**Figure 5:**
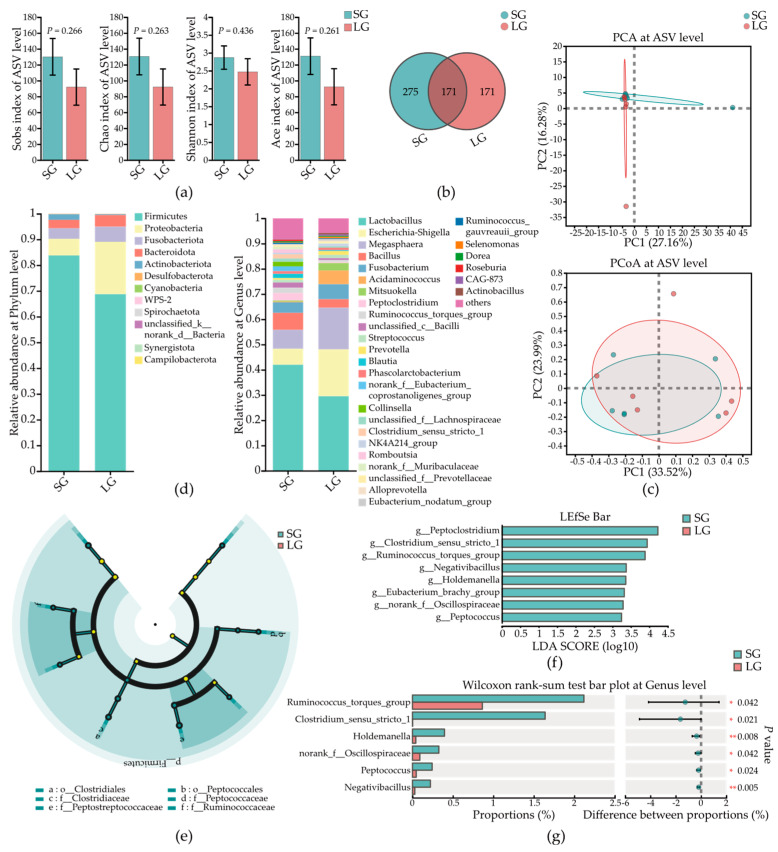
The microbial composition in colonic contents of piglets in the two groups. (**a**) α-Diversity of microbes in colonic contents. (**b**) Venn diagrams of microbes in colonic contents. (**c**) PCA and PCoA results of microbes in colonic contents. (**d**) Community analysis of microbes in colonic contents. (**e**) Cladogram chart of LEfSe result of microbes from Phylum to Family level. (**f**) Bar chart of LEfSe result of microbes at Genus level. (**g**) Top six significantly differentially abundant microbes in colonic contents. SG, standard-birth-weight group; LG, low-birth-weight group; LEfSe, linear discriminant analysis effect size; Data are presented as mean ± SE, and statistical significance was determined by Student’s *t* test (**a**) and the Wilcoxon rank-sum test (**g**); *n* = 6; * *p* < 0.05 and ** *p* < 0.01.

The microbes in colonic contents can affect the metabolism and immunity function of host. Figure 5 shows the microbial composition in colonic contents. There was no significant difference in α-diversity (Figure 5a) (*p* > 0.05), and piglets in SG had more ASVs (Figure 5b). For β-diversity, the colonic mucosa did not have significantly differential microbial composition at ASV level between piglets in two groups based on PCA and PCoA results (Figure 5c). Figure 5d shows the microbial composition at Genus and Phylum level. Linear discriminant analysis effect size (LEfSe) analysis was used to identify the dominant microbial taxa in piglets in the two groups (Figure 5e,f). In the top 50 abundance of microbes at the Genus level, the abundances of six microbial taxa were found to be significantly differential (Figure 5g). In LG, the abundances of seven microbial taxa, which were *Ruminococcus_torques_group*, *Clostridium_sensu_stricto_1*, *Holdemanella*, *norank_f_Oscillospiraceae*, *Peptococcus*, and *Negativibacillus*, were significantly decreased.

### 3.7. Interactions between Colonic Microbes and Glucose-Metabolism-Related Host Genes

**Figure 6:**
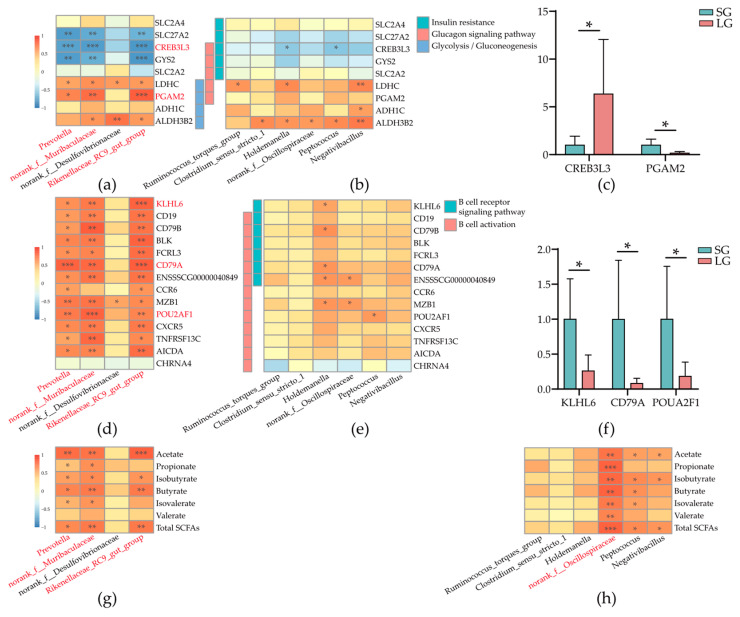
Interactions between colonic microbes and differentially expressed genes or short chain fatty acids and key gene expression validation. (**a**) Interactions between colonic mucosal microbes and glucose-metabolism-related host genes. (**b**) Interactions between microbes in colonic contents and glucose-metabolism-related host genes. (**c**) qRT-PCR analysis for two key genes. (**d**) Interactions between colonic mucosal microbes and B-cell immunity-related host genes. (**e**) Interactions between microbes in colonic contents and B-cell immunity-related host genes. (**f**) qRT-PCR analysis for three key genes. (**g**) Interactions between colonic mucosal microbes and SCFAs. (**h**) Interactions between microbes in colonic contents and SCFAs. The focused microbes or genes were marked with red fonts. qRT-PCR, Quantitative real-time polymerase chain reaction; SCFAs, Short chain fatty acids. Correlations were determined by the Spearman test. Normality of data was determined by the Shapiro–Wilk test. Statistical significance was determined by Student’s *t* test or Mann–Whitney test based on the normality result; *n* = 6; * *p* < 0.05, ** *p* < 0.01, and *** *p* < 0.001.

To investigate the relationships between host genes and microbes in the colon and their potential role in the glucose metabolism in low-birth-weight piglets, we analyzed the correlations between nine DEGs enriched for glucose metabolism and four differentially abundant microbial taxa in colonic mucosa (Figure 6a), in which *Prevotella*, *norank_f_Muribaculaceae*, and *Rikenellaceae_RC9_gut_group* in colonic mucosa were all significantly negatively correlated with three DEGs (*SLC27A2*, *CREB3L3*, and *GYS2*), and *CREB3L3* showed most correlation with these three microbial taxa. Moreover, *norank_f_Muribaculaceae* and *Rikenellaceae_RC9_gut_group* were also significantly positively correlated with *PGAM2*. However, after the analysis of the correlations between DEGs and six differentially abundant microbial taxa in colonic contents (Figure 6b), we found that the correlations were weak. Finally, we used qRT-PCR to validate the key DEGs which were *CREB3L3* and *PGAM2* (Figure 6c), and the expression statuses for these two genes were the same as RNA-seq results.

### 3.8. Interactions between Colonic Microbes and B-Cell Immunity-Related Host Genes

To find bacterial evidence leading to worse immunity in LG piglets, we analyzed the correlations between 14 DEGs enriched in B-cell immunity and four different microbial taxa in colonic mucosa (Figure 6d), in which *Rikenellaceae_RC9_gut_group* in colonic mucosa was highly significantly positively correlated with most of the DEGs, especially *CD79A* and *KLHL6*, and *Prevotella* was also significantly highly positively correlated with *CD79A*. In addition, *norank_f_Muribaculaceae* was highly significantly positively correlated with *POU2AF1*. However, after the analysis of the correlations between DEGs and six differentially abundant microbial taxa in colonic contents (Figure 6e), we found that the correlations were weak. Finally, we used qRT-PCR to validate the key DEGs which were *KLHL6*, *CD79A*, and *POU2AF1* (Figure 6f), and the expression statuses for these two genes were the same as RNA-seq results.

### 3.9. Interactions between Colonic Microbes and Short Chain Fatty Acids

SCFAs are the main metabolites for the energy system of piglets. Therefore, we analyzed the correlations between SCFAs and four differentially abundant microbial taxa in colonic mucosa (Figure 6g) or six differentially abundant microbial taxa in colonic contents (Figure 6h). In our result, *Prevotella* and *norank_f_Muribaculaceae* in colonic mucosa were significantly positively correlated with all SCFAs except for Valerate. Meanwhile, *Rikenellaceae_RC9_gut_group* in colonic mucosa was significantly positively correlated with acetate, isobutyrate, butyrate, and total SCFAs (Figure 6g). Finally, *norank_f_Oscillospiraceae* in colonic contents was highly significantly positively correlated with all SCFAs (Figure 6h).

## 4. Discussion

Piglets in the low-birth-weight group (LG) not only showed significantly lower body weight at 15 days of age (Table S2) but also had significantly lower organ index compared with those in the standard-birth-weight group (SG), which indicates that the growth retardation occurs in low-birth-weight piglets. Intrauterine growth restriction (IUGR), which always occurs with large litter sizes, is the main reason for low-birth-weight piglets [2]. Jinhua pigs had greater reproductive performance with an average of 10 piglets for primiparous sows and an average of 13 live piglets for sows with more than three litters [1]. Therefore, the lower body weight was caused by a large litter size. Meanwhile, a lower organ index, especially for the liver, always indicates worse metabolism and immunity [19]. Specifically, the liver is necessary for nutrient metabolism, especially glucose metabolism, and immunity [6]. In our result, the liver index of piglets in LG showed the lowest *p*-value compared with those in SG, which is compliant with other observations that liver weight is significantly reduced and liver metabolism is damaged in low-birth-weight piglets [6], which indicate that the nutrient metabolism and immunity of piglets in LG are worse.

To further investigate the internal health, we detected the differences in colonic crypt morphology and goblet cell numbers between piglets in the two groups, which can indicate the capacity of secretory function and the health of the mucus barrier [20]. We found that inflammatory factor infiltration, which indicates the inflammation happening in the colon [21], occurred in the colon of piglets in LG. Meanwhile, piglets in LG had a significantly deeper crypt and fewer goblet cells, which means a worse cell secretory function and worse mucus barrier [20], and these worse functions can directly affect the microbiota in the colon, which could further affect early-stage immunity and metabolism [22,23]. Similarly, other findings also show that IUGR piglets always show lower growth, inflammation, and insulin resistance [3], and the growth retardation is always associated with deteriorated intestinal mucosal barrier function [7]. Therefore, piglets in LG have inflammation and a worse mucosa layer, resulting in worse health and growth.

Because LG piglets showed worse immunity and a lower liver index which links to glucose metabolism [6], we analyzed the differences in the colonic transcriptome to further explore the factors affecting the metabolism and immunity of piglets. In our result, almost two-thirds of the differentially expressed genes (DEGs) were downregulated and others were upregulated, and all DEGs only enriched in eight pathways were significantly different based on the KEGG analysis. Because IUGR piglets always have lower growth and insulin resistance [3] and in our result, LG piglets showed lower liver index which is related to glucose metabolism, we focused on the pathways which were related to glucose metabolism, such as glucagon signaling pathway, glycolysis/gluconeogenesis, and insulin resistance. There were nine DEGs enriched in these three pathways with four upregulated genes and five downregulated genes. In the upregulated genes, *CREB3L3* was found to regulate glucose and triglyceride metabolism by synergy with peroxisome proliferator-activated receptor α (PPARα) to regulate Fgf21 expression [24]; the overexpression of this gene is proven to be involved in growth suppression by stimulating systemic lipolysis, hepatic ketogenesis, and insulin sensitivity, which can lead to increased energy consumption to reduce body weight, plasma lipid levels, and glucose levels [25], which can explain why piglets in LG have lower body weight and growth retardation caused by a significantly greater expression of *CREB3L3*. Meanwhile, *CREB3L3* as the negative regulator can inhibit hepatic lipogenesis [26,27], which is in line with our observation that piglets in LG had a lower liver index. Furthermore, *CREB3L3* is involved in acute inflammatory response by regulating *CRP* and *SAP* genes’ expression [28]. Therefore, a greater expression of this gene also indicates that piglets in LG have inflammation. In downregulated genes, *PGAM2* is considered a dominant gene that regulates growth, development, and carcass traits in livestock by enhancing the biosynthesis of amino acids, 5-carbon sugar, and nucleotide precursors [29,30]. A lower expression of *PGAM2* reduces the production of phosphoglycerate mutase which disrupts the energy production of cells [31]. Therefore, the differences in these genes may explain the growth retardation of piglets in LG. Thereafter, IUGR piglets are always characterized by inflammation [3]. Therefore, we further found the DEGs were enriched in the immune system. Go enrichment results showed that DEGs related to immunity were mainly enriched in B cell immunity, and most of them were downregulated in LG piglets. In these genes, *POU2AF1*, which was only enriched in B cell activation, is proven broadly to regulate host defense response genes. A lower expression of *POU2AF1* may lead to a disordered defense system [32]. Meanwhile, it can enable transcription coactivator activity by forming a ternary complex with Oct-1 or Oct-2 transcription factor, which is necessary for B cells’ response to antigens [33], which is in line with our result that piglets in LG with lower expression of *POU2AF1* showed worse immunity. Moreover, *CD79A*, which was enriched in two pathways related to B cell immunity, plays an important role in B cell development and function. Specifically, its protein can participate in B cell receptor formation, along with immunoglobulin light chain and the pre-B-cell antigen receptor (pre-BCR) [34]. Lower levels of *CD79A* protein produced by a lower expression of *CD79A* will reduce B cell development at the pro- to pre B cell transition [35], which indicates that piglets in LG had a worse capacity for B cell immunity. Finally, *KLHL6*, which was only enriched in the B cell receptor signaling pathway, can regulate B-lymphocyte proliferation by *KLHL6*-Roquin2 axis and the inhibition on nuclear factor kappa B (*NF-κB*) [36]. As the tumor suppressor gene, the deficiency of *KLHL6* will promote *NF-κB* activation to drive diffuse large B-cell lymphoma proliferation and promote inflammation [36]. Our results showed that LG piglets had a lower expression of *KLHL6*, which indicates worse inflammation and health. Based on these DEGs, we prove again that LG piglets have inflammation and worse nutrient metabolism.

In addition, short chain fatty acids (SCFAs) are colonic products of microbial carbohydrate fermentation [37], which can regulate gut barrier function, metabolism, and immunity of animals [38]. Specifically, they are sensitive to G protein-coupled receptors to regulate various genes to further affect energy metabolism such as regulating the acetylation of proteins by affecting transcription factors such as SP1 and Foxp3 [39]. Meanwhile, butyric acid is important for animals’ immune system which can promote animals’ immunity by reducing the pro-inflammatory cytokines through inhibiting NF-κB pathways [40,41]. Furthermore, isobutyric acid is observed to regulate lipid and glucose metabolism and signaling in adipocytes’ free fatty acid receptors (FFAR) 2 and 3 [42]. Various observations show that piglets with lower birth weight have a lower production of SCFAs in the intestine [7], which is compliant with our results that LG piglets had fewer intestinal SCFAs, especially butyric acid and isobutyric acid, which indicates LG piglets have worse intestinal health and energy metabolism.

Intestinal microbiota can be seen as another organ for the host, as stable and healthy microbiota support the growth and health of the host. In the intestine, they control the metabolism of the host by their metabolites such as producing SCFAs, and they themselves act as the last barrier to protect the intestine [13]. In addition, when low birth weight occurred, the growth, mammalian embryo/fetus, or organs were impaired during pregnancy, which could affect the microbiota of offspring [15]. Meanwhile, the mucosa layer can strongly affect intestinal microbiota by the glycan repertoire [22], and a worse mucosa layer can directly break the stability of the microbiota [43]. Therefore, to investigate the main factor resulting in the worse health and growth of piglets in LG, we detected the differences in the microbiota in the colon. Although there were no significant differences in the α-diversity and β-diversity of microbiota at the ASV level between piglets in the two groups either in colonic mucosa or contents, LG piglets showed a lower number of ASVs. Liu et al. also obtained a similar result that IUGR piglets had lower OTUs, but there were no significant differences in microbial diversity between IUGR piglets and normal-birth-weight piglets [44]. Lower ASVs may be caused by reduced microbial richness of placenta or delayed gut colonization [45], and not significantly differential microbial diversity, because all piglets were fed breast milk only which affects the microbial diversity extremely [45]. Nevertheless, we still found some differences in the microbiota. In the top 50 abundant microbes, six microbial taxa in colonic digesta and four microbial taxa in colonic mucosa at the Genus level showed significantly differences, and all of them were dominantly colonized in SG piglets. Although not all these dominant bacteria in SG piglets were beneficial microbes, most of them can maintain the metabolism and health of piglets. For example, although *Prevotella* is always associated with inflammation and various diseases, as a commensal (harmless) microbe, it is often abundant in the intestine and female genital tract [46]; therefore, it was dominant in colonic mucosa of SG piglets in our result, and it plays a protective role by training the immune system, deterring invaders, and producing important substances such as SCFAs [47]. In addition, *norank_f_Muribaculaceae* as a beneficial bacterium shows responsibility for cholesterol regulation [48], and it is a prevalent and abundant bacterial component of the gut microbiome of mammals [49]. Similarly, in the significantly differentially abundant microbes in colonic contents, *Clostridium_sensu_stricto_1* was dominantly colonized in SG piglets in our result. Although the function of this bacterium is unknown, it always colonizes in the intestine of breastfed infants during the first month [50], which is in line with our result that SG piglets had a greater abundance of this bacterium. Meanwhile, studies have shown that this bacterium can promote the production of propionate to benefit animals’ immunity [51]. All the microbes above were dominant in SG piglets, and all of them are normal colonized microbes in the intestine or female genital tract. Therefore, a reduction in these bacteria in LG piglets indicate that disrupted microbiota may occur in LG piglets, and these bacterial functions explain why LG piglets had worse immunity and nutrient metabolism, especially SCFA or glucose metabolism.

Finally, to investigate the bacterial evidence leading to worse immunity or nutrient metabolism in LG pigs, we analyzed the correlations between differentially abundant microbes and the chosen DEGs or SCFAs. Firstly, we found that of either DEGs enriched in glucose metabolism or B cell immunity, only three microbial taxa in colonic mucosa containing *Prevotella*, *norank_f_Muribaculaceae*, and *Rikenellaceae_RC9_gut_group* showed significant correlations, which indicates that microbes in colonic mucosa mainly control the glucose and immunity of the host rather than microbes in colonic contents. In specific terms, all of these three microbial taxa showed highly significantly negative correlations with *CREB3L3,* which is involved in growth suppression, hepatic lipogenesis inhibition, and acute inflammatory response [25,27,28], which indicates that lower abundances of these two microbes in LG piglets may remove the inhibition of *CREB3L3,* leading to lower growth performance, lower liver index, and worse immunity. Meanwhile, *Rikenellaceae_RC9_gut_group* can also positively regulate the expression of *PGAM2* to control glucose metabolism. Similarly, other observations also found that *Prevotella* has multiple carbohydrate active enzyme production and polysaccharide utilization loci (PULs) which can help the host to metabolize carbohydrates [52], and *norank_f_Muribaculaceae* has the function of degrading complex carbohydrates [53]. Meanwhile, *Rikenellaceae_RC9_gut_group* can affect the digestion of crude fiber to regulate animals’ carbohydrate metabolism [54], and it can modulate glucose metabolism by promoting glycodeoxycholic acid, alpha-linolenic acid, and glycocholic acid [55], which is in line with our result. Therefore, the reduction in these three microbial taxa may be the main reason for glucose metabolism disorders in LG piglets. In addition, for bacterial evidence leading to worse immunity, these three bacteria showed a highly significantly positive effect on B cell immunity. In our result, *Rikenellaceae_RC9_gut_group* and *Prevotella* can significantly positively affect the expression of *CD79A* to regulate B cell activation and the B cell receptor signaling pathway, and *Rikenellaceae_RC9_gut_group* can also enhance the expression of *KLHL6* to affect the B cell receptor signaling pathway. Similarly, other observations found that *Rikenellaceae_RC9_gut_group* can modulate B-cell differentiation, maturation, and activation by enhancing Tryptophan metabolite levels to stimulate the effects of Tryptophan metabolite receptor aryl hydrocarbon receptor (AhR) [56,57], which is according to our result. Interestingly, there are several findings that *Prevotella* can maintain T-cell immunity by stimulating Toll-like receptors (TLRs) and IL-17 expression [58,59,60], in which TLRs can link humoral immunity and cellular immunity, and TLR signaling can also act on the B-cell or its precursors to affect B-cell differentiation and activation [61], which is in line with our result. What is more, in our result, *norank_f_Muribaculaceae* significantly enhances the expression of *POU2AF1* to modulate B cell activation. *Norank_f_Muribaculaceae* has been reported to regulate homeostatic IgA responses [62], and it can influence both innate and adaptive immune responses via IgA coating [63], which is in line with our result. Therefore, the reduction in these three microbial taxa may lead to the worse B-cell immunity in LG piglets. Finally, for SCFA-production-affected microbes, three microbial taxa in colonic mucosa and one microbial taxon in colonic contents showed significant effects which were *Prevotella*, *norank_f_Muribaculaceae*, *Rikenellaceae_RC9_gut_group*, and *norank_f_Oscillospiraceae*. In our result, all these bacteria were significantly positively correlated with SCFAs’ concentrations which indicate that all of them were SCFA producers, of which *norank_f_Oscillospiraceae* could affect all SCFAs’ production, and the other three microbial taxa mainly influence the production of acetate and butyrate. Based on other observations, these bacteria are also considered as the SCFA producers, which is in line with our observation [64,65,66,67], and *Prevotella* is found to enhance the growth of acetate-producing bacteria, which can further explain our correlation result [68]. Therefore, the reduction in these four bacteria can reduce the production of SCFAs, which leads to worse growth and immunity in LG piglets.

## 5. Conclusions

To sum up, worse nutrient metabolism and immunity appear in low-birth-weight Jinhua piglets. We report the bacterial evidence leading to these phenotypes. In the colon of LG piglets, lower abundances of beneficial microbes in colonic mucosa may lead to worse glucose metabolism and immunity by changing the expressions of glucose-metabolism-related genes or B-cell immunity-related genes. Meanwhile, lower abundances of SCFA producers in the colon of LG piglets may lead to fewer productions of SCFAs, leading to worse energy metabolism and immunity. Therefore, changing intestinal microbiota such as adding probiotics or using fecal microbiota transplantation (FMT) may address the low birth weight of piglets.

## Figures and Tables

**Table 1:** Organ indexes of piglets in two groups.

Parameters	SG	LG	Pooled SD	*p* Value
Heart index (mg/g)	4.80 ± 1.56	3.18 ± 0.81	1.24	0.048
Spleen index (mg/g)	2.05 ± 0.79	1.35 ± 0.33	0.60	0.071
Liver index (mg/g)	28.53 ± 7.50	16.53 ± 0.70	5.33	0.011
Kidney index (mg/g)	4.10 ± 1.12	2.56 ± 0.26	0.81	0.020

Data are presented as mean ± SD; SG: standard-birth-weight group, LG: low-birth-weight group; Normality of data was determined by the Shapiro–Wilk test. Statistical significance was determined by Student’s *t* test or Mann–Whitney test based on the normality result; *n* = 6.

## Data Availability

RNA-seq data can be found in NCBI Sequence Read Archive (SRA) database (Accession Number: PRJNA1018252). The microbiome for the colon can be found in NCBI Sequence Read Archive (SRA) database (Accession Number: PRJNA1017154). Other data that support the findings of this study are available on request from the corresponding author.

## Supplementary Materials

The following supporting information can be downloaded at: https://www.mdpi.com/article/10.3390/microorganisms12071371/s1, Table S1: Composition of the basal diets (%, as fed); Table S2: The body weight of Jinhua newborn piglets; Table S3: Primers of target mRNA genes.
