# Supplementary material for: Disrupted Microbiota of Colon Results in Worse Immunity and Metabolism in Low-Birth-Weight Jinhua Newborn Piglets
Source: Microorganisms. 2024 Jul 4;12(7):1371. doi: 10.3390/microorganisms12071371 (PMC11278573; doi:10.3390/microorganisms12071371)
Supplement: Supplementary file 1 [file microorganisms-12-01371-s001.zip › Table S1.pdf]

Table S1 Composition of the basal diets (%, as fed)

Item	Gestation	Lactation
Ingredient %		
Corn	50	60
Soybean meal	11	15
Wheat bran	17	10
Fish meal	0	2
Soybean skin	5	3
Beet pulp	8	3
Alfalfa	5	3
Premix	4[1]	4[2]
Total	100	100
Nutrient components		
ME (kcal/kg)	2835	3021
NE (kcal/kg)	2002	2174
CP (%)	13.14	15.06
Crude fiber (%)	7.63	5.23
Crude Ash (%)	6.77	6.47
Calcium (%)	0.95	0.94
Available phosphorus (%)	0.35	0.40
Sodium (%)	0.46	0.52
Lysine (%)	0.72	0.86
Methionine (%)	0.20	0.26
Methionine + Cystine (%)	0.45	0.53
Threonine (%)	0.48	0.57
Tryptophan (%)	0.15	0.17
Isoleucine (%)	0.49	0.58
Valine (%)	0.61	0.72
Crude fat (%)	2.82	3.05

[1]Supplied per kilogram of gestation diet: vitamin A, 6,900 IU; vitamin D3, 4,000 IU; vitamin E, 116 IU; vitamin K3, 4.58 mg; thiamine (vitamin B1), 2.12 mg; riboflavin (vitamin B2), 6.34 mg; pyridoxine (vitamin B6), 3.52 mg; vitamin B12, 0.03 mg; biotin, 0.7 mg; pantothenic acid, 22.2 mg; folic acid, 5.0 mg; niacin, 35.2 mg; vitamin C, 100.0 mg; beta-carotene, 2.0 mg; Fe, 120.0 mg; Cu, 15.0 mg; Mn, 60.0 mg; Zn, 95.0 mg; I, 0.7 mg; Se, 0.45 mg; choline chloride, 600 mg and lysine, 1000 mg.

[2]Supplied per kilogram of lactation diet: vitamin A, 6,900 IU; vitamin D3, 4,000 IU; vitamin E, 116 IU; vitamin K3, 4.58 mg; thiamine (vitamin B1), 2.12 mg; riboflavin (vitamin B2), 6.34 mg; pyridoxine (vitamin B6), 3.52 mg; vitamin B12, 0.03 mg; biotin, 0.7 mg; pantothenic acid, 22.2 mg; folic acid, 5.0 mg; niacin, 35.2 mg; vitamin C, 100.0 mg; beta-carotene, 2.0 mg; Fe, 120.0 mg; Cu, 15.0 mg; Mn, 60.0 mg; Zn, 95.0 mg; I, 0.7 mg; Se, 0.45 mg; choline chloride, 600 mg; lysine, 2000 mg and valine, 2000 mg.
