# Supplementary material for: Disrupted Microbiota of Colon Results in Worse Immunity and Metabolism in Low-Birth-Weight Jinhua Newborn Piglets
Source: Microorganisms. 2024 Jul 4;12(7):1371. doi: 10.3390/microorganisms12071371 (PMC11278573; doi:10.3390/microorganisms12071371)
Supplement: Supplementary file 1 [file microorganisms-12-01371-s001.zip › Table S2.pdf]

Table S2 The body weight of Jinhua newborn piglets

Parameters	SG	LG	Pooled SD	P value
Body weight (g)	5333.33 ± 1366.26	3175.00 ± 312.65	991.06	0.011

Data are presented as mean ± SD; SG: standard-birth-weight group, LG: low-birth-weight group; Normality of data was determined by the Shapiro-Wilk test; Significance was determined by the Student's T test and Mann-Whitney test based on the normality result; n = 6.
