# Supplementary material for: Disrupted Microbiota of Colon Results in Worse Immunity and Metabolism in Low-Birth-Weight Jinhua Newborn Piglets
Source: Microorganisms. 2024 Jul 4;12(7):1371. doi: 10.3390/microorganisms12071371 (PMC11278573; doi:10.3390/microorganisms12071371)
Supplement: Supplementary file 1 [file microorganisms-12-01371-s001.zip › Table S3.pdf]

Table S3 Primers of target mRNA genes

Gene	GenBank ID	F-Primer	R-Primer
POU2AF1	NM_001244060.1	CCTCTGTGCTGACCTAC GC	CTGGTGCTCTGGGCCTT
CD79A	NM_001135962.1	ACCTGCCACCATCTTTC TCCTC	AGTGCTGTTGCTGTTGTGTC TG
KLHL6	XM_021069900.1	AATGGGTGAGGGGCTA A	CAGTGTATGCGGAATGTGA
PGAM2	NM_001134968.1	CCATACGCACCCTCTGG A	CCGTCTCCGCCTTGTTG
CREB3L3	XM_021084084.1	CCCTGTCCCTCCTACCC T	AGTTCGGCCTGCTCCTC
GAPDH	NM_001206359.1	TTCCACGGCACAGTCAA G	ACCAGCATCACCCCATTT
